# Causal Associations of Air Pollution With Cardiovascular Disease and Respiratory Diseases Among Elder Diabetic Patients

**DOI:** 10.1029/2022GH000730

**Published:** 2023-06-20

**Authors:** Zhiwei Li, Shiyun Lv, Feng Lu, Moning Guo, Zhiyuan Wu, Yue Liu, Weiming Li, Mengmeng Liu, Siqi Yu, Yanshuang Jiang, Bo Gao, Xiaonan Wang, Xia Li, Wei Wang, Xiangtong Liu, Xiuhua Guo

**Affiliations:** ^1^ Department of Epidemiology and Health Statistics School of Public Health Capital Medical University Beijing China; ^2^ Beijing Municipal Key Laboratory of Clinical Epidemiology Capital Medical University Beijing China; ^3^ Beijing Municipal Health Commission Information Center Beijing China; ^4^ Department of Mathematics and Statistics La Trobe University Melbourne Australia; ^5^ School of Medical Sciences and Health Edith Cowan University Perth Australia; ^6^ National Institute for Data Science in Health and Medicine Capital Medical University Beijing China

**Keywords:** causal inference, cardiovascular disease, respiratory diseases, comorbidity

## Abstract

Extensive researches have linked air pollutants with cardiovascular disease (CVD) and respiratory diseases (RD), however, there is limited evidence on causal effects of air pollutants on morbidity of CVD or RD with comorbidities, particularly diabetes mellitus in elder patients. We included hospital admissions for CVD or RD among elder (≥65 years) diabetic patients between 2014 and 2019 in Beijing. A time‐stratified case‐crossover design based on negative‐control exposure was used to assess causal associations of short‐term exposure to air pollutants with CVD and RD among diabetic patients with the maximum lag of 7 days. A random forest regression model was used to calculate the contribution magnitude of air pollutants. A total of 493,046 hospital admissions were recorded. Per 10 μg/m^3^ uptick in PM_1_, PM_2.5_, PM_10_, SO_2_, NO_2_, O_3_, and 1 mg/m^3^ in CO was associated with 0.29 (0.05, 0.53), 0.14 (0.02, 0.26), 0.06 (0.00, 0.12), 0.36 (0.01, 0.70), 0.21 (0.02, 0.40), −0.08 (−0.25, 0.09), and 4.59 (0.56, 8.61) causal effect estimator for admission of CVD among diabetic patients, corresponding to 0.12 (0.05, 0.18), 0.09 (0.05, 0.13), 0.05, 0.23 (0.06, 0.41), 0.10 (0.02, 0.19), −0.04 (−0.06, −0.01), and 3.91(1.81, 6.01) causal effect estimator for RD among diabetic patients. The effect of gaseous pollutants was higher than particulate pollutants in random forest model. Short‐term exposure to air pollution was causally associated with increased admission of CVD and RD among elder diabetic patients. Gaseous pollutants had a greater contribution to CVD and RD among elder diabetic patients.

## Key Points

- Investigated causal associations of air pollutants on comorbid elderly population
- A stable and accurate effect of air pollution was gained base on large sample sizes
- The effects of gaseous pollutants are stronger than those of PM for the elderly

## 1 Introduction

Cardiovascular disease (CVD) and respiratory diseases (RD) are leading causes of morbidity and mortality worldwide, which cause 330 million and 130 million disease burden, as well as 17.9 million and 3.9 million annual deaths, respectively (Collaborators, 2020; Council, 2019; Disease, 2021; WHO, 2021). Approximately one‐tenth of CVD and one‐third of chronic obstructive pulmonary disease (COPD) exacerbation patients could be attributable to diabetes mellitus (DM) (Castañ‐Abad et al., 2020; Yang et al., 2020). By 2021, there were approximately 141 million patients with diabetes, 26 million patients with CVD, and 172.6 million patients with RD in China. Deaths increase sharply with age (Li et al., 2020). According to China Health Statistics Yearbook, more than 9.84 million and 3.62 million people aged 60 or above hospitalized in 2020 for CVD and RD, respectively.

Individuals with DM have a 4‐6‐fold risk of morbidity and mortality of CVD than those without DM (Cole & Florez, 2020; Zheng et al., 2018). Accelerated by DM due to the same pathophysiologic response, the damage of the pulmonary vascular and alveolus resulting in pulmonary inflammation may be increased, leading to incident RD (Berbudi et al., 2020; Saguil & Fargo, 2020; Visca et al., 2018). However, few is known about the effects of air pollution on patients with comorbidities. Given the heavy burden of CVD with DM (CVD‐DM), as well as RD with DM (RD‐DM), it is essential to identify the modifiable risk factors to provide evidence for prevention in population with comorbidities.

Evidence emerges that air pollution may be one of the common modifiable risk factors for CVD, RD, and DM (Burkart et al., 2022; Liu, Cai, et al., 2022; Ratajczak et al., 2021; Xu, Liu, et al., 2022). Although air pollution has been improved year by year, there are still heavily polluted areas in northern China, such as Beijing (Maji et al., 2020; Zhu et al., 2022). Epidemiologic studies and laboratory experiments suggests that the smaller the particle size, the greater risk to health, that is the effect size of PM_1_ (particulate matter <1 μm in diameter) may be larger than PM_2.5_ (particulate matter <2.5 μm in diameter) and PM_10_ (particulate matter <10 μm in diameter) (Huang et al., 2018; Zou et al., 2017). However, there is limited evidence on causal effect of air pollutants on morbidity of CVD or RD with comorbidities, particularly DM in elder patients. We hypothesizes that air pollutants have greater effects on CVD‐DM patients, as well as RD‐DM patients in the elderly.

Due to the inherent unobserved confounding, the regression coefficients always reflect the association rather than causal effect, which can be estimated by causal inference models such as instrumental variable and difference‐in‐difference (Streeter et al., 2017; Williams et al., 2019). But it is difficult to obtain unbiased estimation, whereas negative control exposure can accurately estimate the causal effect by comparing the effects of post‐outcome exposures and pre‐outcome exposures on outcomes.

Therefore, this study aimed to estimate the causal association of short‐term exposure to particulate air pollutants (PM_1,_ PM_2.5_, and PM_10_), and gaseous air pollutants (NO_2_, SO_2_, CO, O_3_) with morbidity of CVD‐DM and RD‐DM in the elderly. Furthermore, we ranked effect sizes to determine the predominant air pollutants for CVD‐DM and RD‐DM.

## 2 Material and Methods

### 2.1 Data Collection

We collected admission data on comorbid patients older than 65 years between 2014 and 2019 from the Beijing Center for Big Data and Policy Research in Health, a government department responsible for statistical work such as collecting, quality control, collating and aggregating data from health care institutions across Beijing (Health, 2022). We obtained daily amounts of admission data for CVD‐DM and RD‐DM from this institution. The study population consisted of patients older than 65 years to assess the effects on a vulnerable population.

Admission diagnosis was determined by International Classification of Diseases 10th Revision (ICD‐10) provided at the time of admission for CVD or RD. Total CVD was defined as the sum of the number of people with each subtype, including ischemic heart disease (IHD, I20 to I25), ischemic stroke (IS, I63) and unspecific stroke disease (USS, I64 to I69). Total RD was defined as the sum of chronic obstructive pulmonary disease (COPD, J44) and Lower respiratory tract infections (LR, J12 to J22). Type 2 DM was coded as E11. For ease of presentation all respiratory and cardiovascular subtypes of disease appearing in the text represent comorbid diabetes. For example, IHD indicates IHD with comorbid diabetes, and COPD denotes COPD with comorbid diabetes.

Air pollution data were obtained from the ChinaHighAirPollutants (CHAP) data set, a long‐term, full‐coverage, 1 km spatial resolution and high‐quality data set of ground‐level air pollutants in China. CHAP was generated from big data (e.g., ground‐based measurements, satellite remote sensing products, atmospheric reanalysis, and model simulations) using artificial intelligence considering the spatial and temporal heterogeneity of air pollution (weijing, 2022a).

The daily air pollutants of Beijing were collected from the CHAP data set include PM_1_ (μg/m^3^, PM_2.5_ (μg/m^3^), PM_10_ (μg/m^3^) and ozone (O_3_, μg/m^3^). Since the time scale of admission data is days, but the minimum data scale of nitrogen dioxide (NO_2_, μg/m^3^), sulfur dioxide (SO_2_, μg/m^3^) and carbon monoxide (CO, mg/m^3^) in CHAP data is months, which does not match with our admission data (weijing, 2022b, 2022c, 2022d). So we collected daily NO_2_, SO_2_ and CO data from the Beijing Environmental Protection Bureau (BEPB) to replace the NO_2_, SO_2_ and CO data from CHAP data. The BEPB, one of the first professional environmental monitoring institutions to acquire national‐level metrological certification, has been awarded a certificate of conformity for metrological certification by the China Technical Supervision Bureau. Air pollution data were collected and monitored according to the standard methods defined in the Ambient Air Quality Standard GB 3095‐2012 (China, 2016). The temperature (°C) and relative humidity (%) were gathered from the China Meteorological Data Sharing Service Online (online, 2022).

### 2.2 Statistic Analysis

The association between PM and gas pollutants was calculated by Spearman rank correlation. A time‐stratified case‐crossover design was used to assess the magnitude of the effects of different air pollutants on comorbid patients, which was one of the most common designs in the assessment of the health effects of air pollution (Wu et al., 2021; Xu, Shi, et al., 2022). Under this design, the case day was defined as the date of admission and each subject served as his or her control in the same stratum (e.g., 1 month). The control day was defined as any other identical day of the week in the same year and month as the case day, which yielded a designated reference window of 3 or 4 days. This design was done to control for potential confounding factors, including individual covariates, day of the week, seasonality, and time trend (Lu et al., 2022). Conditional Poisson regression was used to perform the time‐stratified case‐crossover study. The model as follow:

$$\mathrm{Log}\;\left(E\left(Y\right)\right)=a+\beta_{0}\times expo+\beta_{1}\times stratum+\beta_{2}\times PCA+\beta^{T}\times covariates$$

where E(Y) is the expectation of daily admissions for patients with comorbidities; a is the intercept; expo is different air pollutant in our study including PM_1_, PM_2.5_, PM_10_, SO_2_, NO_2_, CO, O_3_ with its coefficient $\beta_{1}$; stratum denotes the time window by grouping the same day of the week within each month of each year; $\beta_{2}$; PCA is a composite latent variable substituted other pollutants using principal component analysis to address collinearity among air pollutants; $\beta^{T}$ denotes the coefficients of covariates, and covariates stands for a cross‐basis matrix of temperature and relative humidity to control for both exposure‐response and lag‐response effects using *dlnm* package.

We reported percentage change (PC) followed by corresponding 95% confidence interval (95% CI) to quantify the magnitude of the association effect. Briefly it is calculated from the regression coefficients of air pollutants in Conditional Poisson regression. The formula is given below.

$$PC=\left[\exp\left(\beta_{0}*∆c\right)-1)\right]*100$$

where $\beta_{0}$ is consistent with $\beta_{0}$ in the Conditional Poisson regression model described above. ∆c is the unit increase number of air pollution concentration. In our study, ∆c is set to 10 μg/m^3^ for PM_1_, PM_2.5_, PM_10_, SO_2_, NO_2_, O_3_, and 1 mg/m^3^ for CO to get an appropriate effect value.

Considering that the effects of air pollutants on CVD‐DM or RD‐DM were more accurate in multi‐pollutant models, we presented the multi‐pollutant model results as the primary outcome. Single lag and moving lag were used to explore the lag effect of air pollutants. Lag1 indicates that the number of comorbid admissions on the current day is influenced by the previous day's air pollution concentration; Lag01 means that the number of comorbid admissions on the current day is influenced by the average of the current day and the previous day's air pollution concentration. Since we only focused on the short‐term effects of air pollutants, the maximum lag we chose is 7 days (Li et al., 2022a; Liu, Li, et al., 2022).

Next, to achieve a better understanding of the burden of CVD‐DM and RD‐DM attributable to air pollutant exposure, we used conditional Poisson regression‐based attributable numbers (AN) and population attributable fractions (PAF). The detailed descriptions of the AN and PAF calculations have been presented in our previous publications (Li et al., 2022b). We used different air quality standards, including those of China and the World Health Organization, as reference concentrations. Since neither China nor WHO had a standard for PM_1_, we calculated the mean and quartile of PM_1_ as the reference concentration during the study period.

Following that, a random forest regression model was used to calculate the magnitude of the contribution of particulate and gaseous pollutants to the effect of patients with CVD‐DM and RD‐DM. A random forest is a classifier containing multiple decision trees, and the output class is determined by the plurality of the output classes of the individual trees (Gariazzo et al., 2020). In our study, we put 7 different air pollutants and other confounding into the random forest model at the same time including temperature, relative humidity, day of week, holiday and time trends. After that we calculated the mean square error (MSE) and node purity for different types of air pollutants, the larger the MSE or node purity, the greater the effect of air pollution on comorbidity patients. Please see the attached material for the specific process of building the random forest model (Figures S1 and S2 in Supporting Information S1).

Finally, we used the negative‐control exposure based on a time‐series studies (NCETS) method to explore the causal association between air pollutants and patients with comorbidities. This was an improved method of causal inference, proposed by Xue et al. in 2021 and it can effectively eliminate unobserved confounders using an after‐outcome exposure as a negative‐control exposure (Yu et al., 2021). The proposed NCETS model was implemented in an R package called NCETS, freely available on GitHub. Please see the attached material for the specific process of building the NCETS model (Figure S3 in Supporting Information S1).

### 2.3 Sensitivity Analysis

A series of sensitivity analyses were conducted to confirm the robustness of the results. First, single pollutant models of the conditional Poisson regression were also built to assess the effects. Second, we calculated E‐value to assess the unmeasured confounding bias. The current results are considered robust if the RR is less than the corresponding E‐value (VanderWeele & Ding, 2017). Third, in the random forest model, mtry refers to the optimal number of variables to be used for the binomial tree in a given node. We use the default value (mtry = 16) and the optimal mtry value (mtry = 33) to test the stability of the random forest results. Finally, in causal inference estimation, confidence intervals provide two different methods of estimation. The default method is “normal,” where the confidence interval is derived by calculating the covariance matrix. In addition, the NCETS model also provides a resampling method (method = “bootstrap”) to estimate confidence intervals. We use both methods to estimate the confidence intervals for the causal effects.

All statistical analyses were conducted in R software (version 4.2.0) using the tidyverse, gnm, dlnm, randomForest and NCETS packages. The statistical tests were two‐sided, and *P* < 0.05 was considered statistically significant.

## 3 Result

### 3.1 Descriptive Results

A total of 493,046 patients with comorbid diabetes were admitted during the study period, including 375,817 patients with CVD‐DM and 117,229 patients with RD‐DM. Patients with IHD accounted for 54.33% among CVD‐DM individuals, and LR patients accounted for 65.86% among RD‐DM individuals. During the study period, PM_10_ has the highest average concentration of 64.39 μg/m^3^ and SO_2_ has the lowest average concentration of 9.81 μg/m^3^ (Table 1). All air pollutants were above the WHO reference levels, while PM_2.5_, PM_10_, NO_2_, and CO were above the Chinese government reference levels (Table 2). The correlation heat map results showed that there were moderate correlations between different air pollutants and meteorological factors, where there were strong correlations between PM_1_, PM_2.5_, PM_10_, and CO (correlation coefficient >0.80) (Figure S4 in Supporting Information S1).

**Table 1:** Distribution of Admission, Air Pollution and Meteorological Variables Between 2014 and 2019, in Beijing, China

Daily variables	Total cases (%)	Guide			Mean	Standard deviation	Minimum	Quartile			Maximum
		China	WHO2005	WHO2021				P_25_	P_50_	P_75_	
Admission											
CVD‐DM	375,817 (100.00)	–	–	–	171.61	76.70	1	90	180	229	467
IHD‐DM	204,187 (54.33)	–	–	–	93.28	51.41	1	46	93	133	290
IS‐DM	121,605 (32.36)	–	–	–	55.53	22.03	1	35	58	72	126
USS‐DM	50,025 (13.31)	–	–	–	22.85	11.76	1	12	24	31	64
RD‐DM	117,229 (100.00)	–	–	–	53.50	23.95	3	33	53	70	147
COPD‐DM	40,018 (34.14)	–	–	–	18.26	8.32	2	12	18	24	50
LR‐DM	77,211 (65.86)	–	–	–	35.24	19.02	3	20	33	48	110
Air pollution											
PM_1_ (ug/m^3^)	–	–	–	–	34.83	20.68	6.83	18.54	29.69	46.28	104.48
PM_2.5_ (ug/m^3^)	–	75	25	15	62.79	46.57	8.60	27.28	50.07	85.66	242.53
PM_10_ (ug/m^3^)	–	150	50	45	94.39	53.52	22.53	53.57	82.73	122.17	291.97
NO_2_ (ug/m^3^)	–	80	–	25	45.05	17.87	15.93	31.85	41.80	54.86	99.86
SO_2_ (ug/m^3^)	–	150	20	40	9.81	9.39	2.32	3.45	6.37	12.02	52.80
CO (mg/m^3^)	–	4	–	4	1.00	0.62	0.30	0.58	0.84	1.17	3.67
O_3_ (ug/m^3^)	–	160	100	100	59.54	33.45	7.33	32.51	55.59	81.48	145.93
Meteorological factor											
Temperature (°C)	–	–	–	–	13.02	11.35	−10.55	2.15	14.41	23.39	30.77
Humidity (%)	–	–	–	–	53.75	17.77	20.83	38.93	53.47	69.20	86.71

*Note.* IHD: Ischemic heart disease; IS: Ischemic stroke; USS: Unspecific stroke disease; COPD: Chronic obstructive pulmonary disease; LR: lower respiratory tract infections.

**Table 2:** The Attributable Risk Due To Different Air Pollution for CVD‐DM and RD‐DM Admissions

Pollution	Index	Guide^a^	CVD‐DM		RD‐DM	
			Attributable number (95% CI	Population Attributable fraction (95% CI)	Attributable number (95% CI)	Population Attributable fraction (95% CI)
PM_1_	Mean	36	4131.01 (2555.28–5739.04)	1.1 (0.68–1.53)	739.73 (−173.1–1686.84)	0.63 (−0.15–1.44)
	P_25_	18	8379.07 (5181.85–11,643.25)	2.23 (1.38–3.1)	1487.86 (−348.04–3394.18)	1.27 (−0.3–2.9)
	P_50_	30	4763.35 (2946.28–6617.85)	1.27 (0.78–1.76)	830.9 (−194.42–1894.9)	0.71 (−0.17–1.62)
	P_75_	48	2770.86 (1713.96–3849.43)	0.74 (0.46–1.02)	413.1 (−96.67–941.99)	0.35 (−0.08–0.8)
PM_2.5_	China	75	4305.64 (3233.02–5401.23)	1.15 (0.86–1.44)	1332.7 (773.96–1914.24)	1.14 (0.66–1.63)
	WHO2005	25	1977.29 (10498.14–17529.21)	3.72 (2.79–4.66)	4958.37 (2881.04–7118.58)	4.23 (2.46–6.07)
	WHO2021	15	18079.36 (13577.27–22676.86)	4.81 (3.61–6.03)	6432.54 (3736.63–9237.43)	5.49 (3.19–7.88)
PM_10_	China	150	1443.79 (1081.4–1814.65)	0.38 (0.29–0.48)	417.3 (227.84–614.96)	0.36 (0.19–0.52)
	WHO2005	50	11926.39 (8951.3–14956.57)	3.17 (2.38–3.98)	3833.31 (2100.58–5625.23)	3.27 (1.79–4.8)
	WHO2021	45	15369.29 (11534.72–19275.23)	4.09 (3.07–5.13)	4389.92 (2405.35–6442.63)	3.74 (2.05–5.5)
CO	China^b^	4	310.08 (228.77–393.43)	0.08 (0.06–0.1)	129.96 (81.25–180.83)	0.11 (0.07–0.15)
SO_2_	China	150	NA	NA	NA	NA
	WHO2005	20	347.05 (−114.08 to 817.46)	0.09 (−0.03 to 0.22)	459.56 (10.11–940.82)	0.39 (0.01–0.8)
	WHO2021	40	150.7 (−49.56 to 354.83)	0.04 (−0.01 to 0.09)	244.37 (5.38–499.51)	0.21 (0–0.43)
O_3_	China	160	NA	NA	NA	NA
	WHO2005^c^	100	1510.12 (248.87–2786.68)	0.40 (0.07–0.74)	3.90 (−719.13 to 744.75)	0.00 (−0.61 to 0.64)
NO_2_	China	80	2133.96 (1898.08–2372.86)	0.57 (0.51–0.63)	600.83 (464.34–740.75)	0.51 (0.4–0.63)
	WHO2021	25	35736.29 (31698.9–39847.68)	9.51 (8.43–10.6)	11648.35 (8954.57–14439.31)	9.94 (7.64–12.32)

^a^ CO concentration unit is mg/m^3^; PM_1_, PM_2.5_, PM_10_, SO_2_, NO_2_, O_3_ concentration unit is ug/m^3^.

^b^ China and WHO 2021 on CO standards are the same.

^c^ WHO2005 and WHO2021 standards on ozone are the same.

### 3.2 Association of Air Pollution With Morbidity of CVD‐DM and RD‐DM

**Figure 1:**
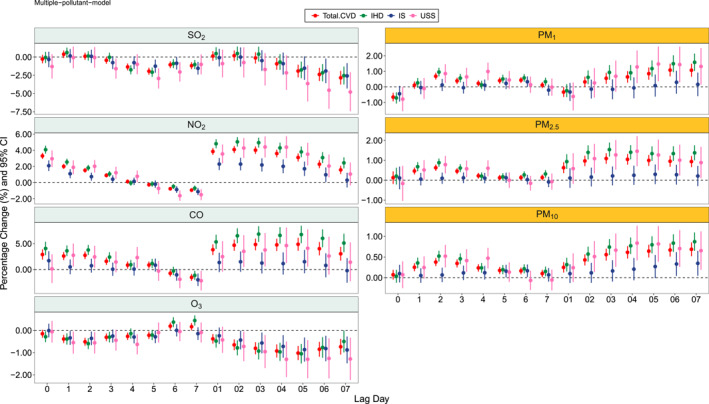
The percentage change (PC) for CVD‐DM patients in 7 air pollutants with different lag structure in multiple model (temperature, relative humidity, and the other 6 air pollutants controlled in the model). Note: PC for PM_1_, PM_2.5_, PM_10_, SO_2_, NO_2_, and O_3_ were per 10 μg/m^3^ increase and 1 mg/m^3^ for CO.

**Figure 2:**
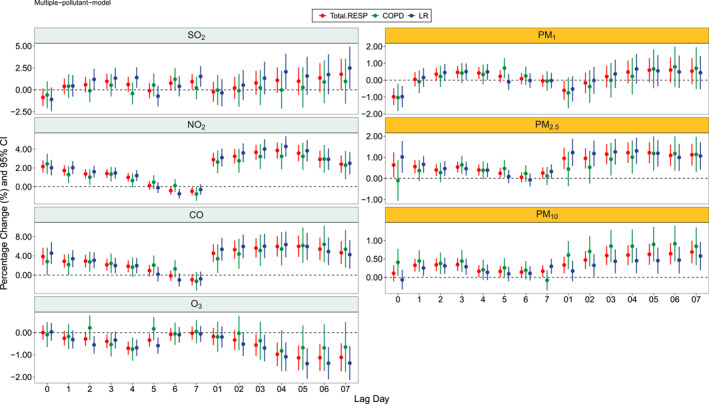
The percentage change (PC) for RD‐DM patients in 7 air pollutants with different lag structure in multiple model (temperature, relative humidity, and the other 6 air pollutants controlled in the model). Note: PC for PM_1_, PM_2.5_, PM_10_, SO_2_, NO_2_, and O_3_ were per 10 μg/m^3^ increase and 1 mg/m^3^ for CO.

Figures 1 and 2 showed the PC in each air pollutants for CVD‐DM and RD‐DM patients with different lags in multiple pollutant model. There was a positive association between air pollutants, especially particulate pollutants in which statistically significant associations were found in PM_1_, PM_2.5_, and PM_10_. The lag day that the maximum effect occurred varied for different pollutants. For example, for CVD‐DM patients, the maximum PC values were 1.08 (0.68, 1.49), 1.09 (0.83, 1.36) and 0.69 (0.52, 0.85) for PM_1_ at lag 06, PM_2.5_ at lag 03 and PM_10_ at lag 07, respectively. For patients with RD‐DM, the PC values were greatest at lag 06, lag 03, and lag 07 for PM_1_, PM_2.5_, and PM_10_, corresponding to 0.60 (−0.14, 1.35), 1.15 (0.67, 1.63), and 0.69 (0.38, 1.00) in PC value.

We further calculated the attributable risk of air pollutants on CVD‐DM and RD‐DM admissions based on China and WHO air quality guideline. More admissions will be avoided, using the latest WHO 2021 air quality guideline as the standard. In Table 2, the maximum attributable fraction among all pollutants is NO_2_ using WHO 2021 air quality guideline as the reference, which was 9.51% (95% CI: 8.43%–10.60%) of CVD‐DM and 9.94% (95% CI: 7.64%–12.32%) of RD‐DM patients, respectively.

### 3.3 Comparison of Contribution of Particular Matter and Gaseous Pollutants to CVD‐DM and RD‐DM Patients

Table 3 showed the contributions of different air pollutants in the random forest regression model when the best mtry (mtry = 33) was used. The results showed that the effect size of gaseous pollutants was always larger than that of particulate pollutants (the values of MSE and node purity were both the highest in gaseous pollutants), regardless of morbidity of CVD‐DM or RD‐DM. For instance, in CVD‐DM, the MSE of gaseous pollutants is 12.06 and 12.14 which are higher than the MSE of particulate pollutants. Similar results were also found in RD‐DM And the explainable variances of the models were both above 85%.

**Table 3:** The Contribution of Gaseous and Particulate Pollutants in Random Forest Model for CVD‐DM and RD‐DM Patients With Different Mtry Value

mtry^a^	Outcome	Pollution type	MSE^b^	Node purity	Explained variance
33	CVD‐DM	Gaseous^c^	12.06	51,391.65	86%
		Particulate^d^	8.17	38,429.43	
	RD‐DM	Gaseous	8.41	6599.18	85%
		Particulate	7.21	4359.36	
16	CVD‐DM	Gaseous	12.14	80,720.8	86%
		Particulate	8.23	61,470.88	
	RD‐DM	Gaseous	10.75	11,330.48	84%
		Particulate	8.67	7221.05	

^a^ The optimal mtry is the mtry corresponding to the minimal Out Of Bag Error in model (mtry = 33), and the default mtry is the number of variables divided by 3 (mtry = 16).

^b^ Mean Square Error.

^c^ SO_2_, NO_2_, CO, O_3_ are included in gaseous pollution.

^d^ PM_1_, PM_2.5_, PM_10_ are included in particulate pollution.

### 3.4 The Causal Effect of Different Pollutants on Morbidity of CVD‐DM and RD‐DM

**Figure 3:**
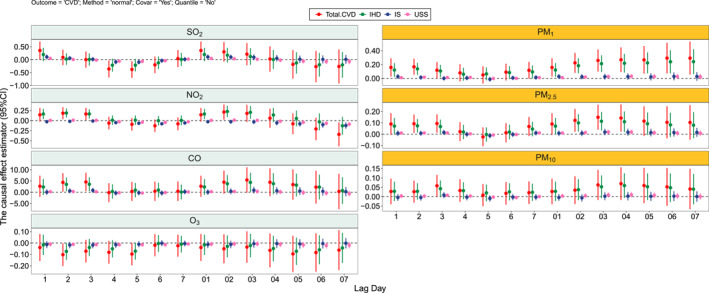
Causal effects of different air pollutants for total and specific CVD‐DM patients using “normal” method.

**Figure 4:**
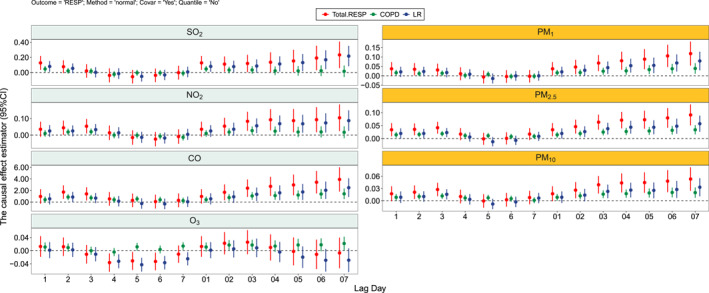
Causal effects of different air pollutants for total and specific RD‐DM patients using “normal” method.

Figures 3 and 4 showed the causal effect estimator in seven air pollutants in the NCETS model. “Normal” method which means calculate the covariance matrix was used to estimate the confidence interval. There was a positive causal effect between air pollutants except O_3_ and hospital admissions in patients with CVD‐DM and RD‐DM. In the stratified disease analysis, the causal effect of air pollutants was stronger in IHD‐DM patients than in IS‐DM and USS‐DM patients, and the results of the stratified analysis in RD‐DM patients showed that the causal effect of air pollutants was stronger in LR‐DM patients than in COPD‐DM patients.

### 3.5 Sensitivity Analysis

The inclusion or non‐inclusion of other air pollutants in the model only affected the size of the air pollutant of interest effect and did not result in a statistically significant inversion (Figures S5 and S6 in Supporting Information S1). The E‐value calculated for the total and cause‐specific disease in different air pollutant did not exceed the original RR (Tables S1 and S2 in Supporting Information S1). The results were very close when we used the default mtry (mtry = 16) in random forest regression model, comparing to the optimal mtry value (mtry = 33) (Table S3 in Supporting Information S1). In causal effect estimate, changing the method to “bootstrap” to estimate the confidence interval did not substantially change the effect estimates (Figures S7 and S8 in Supporting Information S1). For more detailed results, please see the attachment.

## 4 Discussion

To our knowledge, this was the first study to compare the importance and evaluate causal effects of air pollution for CVD‐DM and RD‐DM patients in the elderly. In addition to statistical associations, we also found that exposure to air pollutants was causally associated with increased hospital admissions for CVD‐DM and RD‐DM patients in the elderly. Gaseous air pollutants had higher effects than particulate air pollution both for CVD‐DM and RD‐DM patients in the elderly. The application of tighter guidelines for air quality standards would significantly reduce the number of patients with CVD‐DM and RD‐DM in the elderly. Our findings added to the body of research on air pollutants effect on CVD‐DM and RD‐DM morbidity.

In the present study, we observed more acute and larger effects of air pollutants on CVD‐DM and RD‐DM among elder patients than CVD and DR without combined DM reported by other epidemiological studies (Rahman et al., 2021; Renzi et al., 2022). A study from Italy reported an effect of PM_2.5_ and PM_10_ on CVD patients at lag05, while Yu et al. found that the effect of PM_2.5_, PM_10_, and NO_2_ on hospital admissions in RD patients was effective at lag07, lag07, and lag03, which were more delayed than our study (Hua et al., 2022; Stafoggia et al., 2022). Some studies have reported that the PC is 1.04% for PM_10_ and 1.28% for NO_2_ on CVD patients, and 1.42% for NO_2_ on RD patients, which were weaker than our study (Phosri et al., 2019). However, Adebayo‐Ojo et al. in South Africa found an effect of PM_10_ on CVD patients of 2.1% and RD patients of 1.9%, which were higher than the CVD‐DM and RD‐DM patients in our study (Adebayo‐Ojo et al., 2022). This may be related to the composition of the particulate matter. Particulate matter is a complex mixture, and the toxicological properties of different chemical components may show different effect sizes in different areas depending on time and location factors (Yang et al., 2018). The relationship between CVD‐DM and RD‐DM patients and SO_2_ changed to negatively associated or unassociated after adjusting for other pollutants, which could be explained by the possible confounding of the SO_2_ effect with other air pollutants, and also by covariance in the regression model, as pointed out in other literature (Liu, Tian, et al., 2018; Peng et al., 2008). The negative association between ozone and comorbid patients may be due to the differences in pollutant levels and pollution distribution of various geographical locations.

Causal effects were significantly positive between particulate air pollution (PM_1_, PM_2.5_, PM_10_) and gaseous air pollutants (NO_2_, SO_2_, CO), with CVD‐DM and RD‐DM, which was the same as other studies (Qiu et al., 2020). Yazdi et al. found PM_2.5_ was associated with increased admissions with CVD and NO_2_ was associated with increased admissions for RD (Yazdi et al., 2022). A study in Thailand reported PM_10_, SO_2_, NO_2_, and CO were associated with cardiovascular and respiratory admission (Phosri et al., 2019). Ugalde‐Resano et al. also reported positive associations for PM_10_, PM_2.5_, NO_2_ and CO in cardiovascular and respiratory disease (Ugalde‐Resano et al., 2022). The pathogenic mechanisms of air pollutants in patients with comorbidities have been proposed. First, elevated glucose levels directly increased viral proliferation and decreased cardioprotective effects via the production of mitochondrial reactive oxygen species and activation of hypoxia‐inducible factor 1α (Xie et al., 2017; Ye et al., 2020). It was reported that high glucose concentrations increased the intracellular levels of methylglyoxal and glycated proteins that in turn actuated the unfolded protein response and triggered inflammatory and prothrombotic pathways (Irshad et al., 2019). Second, Glycated apolipoprotein A‐I formed during hyperglycemia increased the morbidity of cardiovascular disease and respiratory disease by modifying structure, decreasing lipid‐binding ability, preventing cholesterol efflux from macrophage and impairing anti‐inflammatory function (Edmunds et al., 2019; Liu, Ji, et al., 2018).

We found that gaseous pollutants had a higher effect than particulate pollutants in elderly patients with comorbidities when air pollutants were classified and incorporated into random forest model, which was stable regardless of changing the value of the parameter mtry and the disease stratification analysis. This may have a special relationship with the high level of air pollution in our study area, Beijing. In 2013, Beijing published the “Beijing Clean Air Action Plan 2013–2017,” which focused on the goal of controlling the annual average PM_2.5_ concentration in Beijing at around 60 μg/m^3^ in 2017 (Government, 2013). With the closure of some of Beijing's heavily polluting factories and the use of clean energy, PM_2.5_ and PM_10_ concentrations have dropped significantly. However, with the development of the economy, the number of motor vehicles in Beijing continues to increase. As of 2019, there are 6.365 million motor vehicles in Beijing, an increase of 281,000 over the previous year, which significantly increases the emissions of traffic‐related air pollutants, such as CO and NOx (Government, 2019). As the associations between pollutants and CVD or RD were different at higher levels of exposure, the combination of the above causes leads to an increase in the effect of gaseous pollution and a decrease in the effect of particulate pollutants (Chen et al., 2020).

We found different effects of air pollutants on different disease subtypes. For example, among CVD‐DM patients, we failed to observe significant associations between particulate pollutants and IS patients, which was consistent with previous studies. A study based on a population covering 280 million urban workers' basic medical insurance enrollees in China showed that the effect of PM_2.5_ on hospital admissions of IS patients was only significant in single‐pollution model. When adjusted separately for SO_2_, NO_2_, and CO, the effect of PM_2.5_ on hospital admissions for IS patients was not statistically significant (Tian et al., 2018). Wellenius and colleagues based on Medicare recipients aged 65 years and older in 9 US cities found that the effect of PM_10_ on IS onset was not statistically significant at lag1 and lag2. After imputing the data for PM_10_, there was still no significance (Wellenius et al., 2005). Similar findings were found in studies by other scholars (Liu et al., 2017).

Our study has several strengths. First, we estimated causality on the basis of association. We estimated the effect magnitude of the association of air pollutants on comorbid patients using conditional Poisson regression based on a case‐crossover design. After that, we estimated causal effect sizes for air pollutants using a causal inference model, which allows us to observe the relationship between air pollutants and comorbid patients in a more integrated way. Second, the causal model we used was the one improved by Yu et al., in 2021. Because most environmental data lack sufficient covariates, traditional causal inference methods that do not adequately adjust for confounders will inevitably lead to the generation of residual confounding. The NCETS model proposed by Yu and colleagues uses post‐outcome exposures as negative control exposures to effectively eliminate unobserved confounders. Third, the study population was focused on CVD‐DM and RD‐DM patients in the elderly, as most people have comorbidities and diabetes can enhance or worsen cardiovascular and respiratory disease. Age has been increasingly regarded as a potential factor that modifies PM‐morbidity associations. Fourth, we used multiple sensitivity analyses to verify the stability of our results. In our study, we used Poisson regression based on a case‐crossover design, random forest, and NCETS model, respectively. In each model, we used different sensitivity analyses. And the results of the sensitivity analyses were consistent with the main results, which made the conclusions we drew more reliable. Fifth, our patient data are highly reliable. We collected data on comorbid patients from Beijing Center for Big Data and Policy Research in Health, which is responsible for the statistical collection of inpatient data from all hospitals above the second level in Beijing. The representative sample of cardiovascular disease and respiratory disease with diabetes patients in Beijing gives a strong statistical power for the estimate.

Several limitations should be noted. First, in the protocol with Beijing Center for Big Data and Policy Research in Health, we were not allowed to collect patients with CVD or RD alone at the time of data collection, which allowed us to compare our results only with those of other studies. The differences in regions, susceptible populations and pollutant concentrations between studies may have caused our comparative results to be less than accurate. We will continue to negotiate and collaborate with Beijing Center for Big Data and Policy Research in Health to add patients with CVD or RD without combination in future studies. Second, not all air pollution data are from high‐resolution satellite data. Because SO_2_, NO_2_ and CO in the CHAP data set did not match the admission data on the time scale, we used fixed‐site based monitoring data as a substitute, which introduced some heterogeneity in the air pollution data. For future studies we will look for air pollution data with uniform sources, higher quality, and higher resolution. Third, we used average concentration for personal exposure in Beijing, which may result in exposure errors and underestimation of the associations between ambient air pollution and diseases. Fourth, caution should be taken once extrapolating our results to other areas because Beijing is only a representative city with high levels of air pollution.

## 5 Conclusion

In conclusion, exposure to air pollution was causally associated with increased hospital admissions for CVD‐DM and RD‐DM in the elderly. Elder patients with CVD‐DM and RD‐DM were more susceptible to gaseous pollutants. Our findings added evidence on air pollutants with CVD‐DM and RD‐DM morbidity. The relative importance of gaseous pollutants reminded the public to take measures to reduce counterpart exposures and policymakers to focus on the management of counterpart pollutants.

## Conflict of Interest

The authors declare no conflicts of interest relevant to this study.

## Supporting information

Supporting Information S1

## Data Availability

The raw/processed patient data are not available to the public due to the Information Center of Beijing Municipal Health Commission's data policy. Air pollution data from the CHAP data set including PM_1_, PM_2.5_, PM_10_, and O_3_ can be found online at https://weijing-rs.github.io/product.html (Wei & Li, 2019, 2020, 2022; Wei et al., 2020). Air pollutant data of NO_2_, SO_2_, and CO data, and meteorological data are available at https://quotsoft.net/air/ and http://data.cma.cn, which acquirement steps for downloading can be found in the supplementary “Text S3 in Supporting Information S1. Data download steps.” Our manuscript does not contain any individual person's data in any form (including any individual details, images or videos), so written informed consent was not required.
