# Supplementary material for: Causal Associations of Air Pollution With Cardiovascular Disease and Respiratory Diseases Among Elder Diabetic Patients
Source: Geohealth. 2023 Jun 20;7(6):e2022GH000730. doi: 10.1029/2022GH000730 (PMC10282596; doi:10.1029/2022GH000730)
Supplement: Supplementary file 1 — Supporting Information S1 [file GH2-7-e2022GH000730-s001.docx]

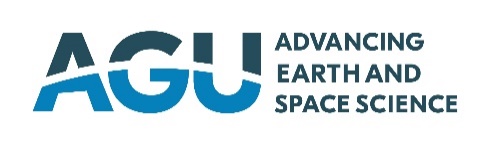


*GeoHealth*

Supporting Information for

**Causal associations of air pollution with cardiovascular disease and respiratory diseases among elder diabetic patients**

Zhiwei Li^1,2^, Shiyun Lv^1,2^, Feng Lu^3^, Moning Guo^3^, Zhiyuan Wu^1,2^, Yue Liu^1,2^, Weiming Li^1,2^, Mengmeng Liu^1,2^, Siqi Yu^1,2^, Yanshuang Jiang^1^, Bo Gao^1,2^, Xiaonan Wang^1,2^, Xia Li^4^, Wei Wang^5^, Xiangtong Liu^1,2*^, Xiuhua Guo^1,2,5,6*^

^1^ Department of Epidemiology and Health Statistics, School of Public Health, Capital Medical University, Beijing, 100069, China;

^2^ Beijing Municipal Key Laboratory of Clinical Epidemiology, Capital Medical University, Beijing, 100069, China;

^3^ Beijing Municipal Health Commission Information Center, Beijing, 100034, China;

^4^ Department of Mathematics and Statistics, La Trobe University, Melbourne, 3086, Australia;

^5^ School of Medical Sciences and Health, Edith Cowan University, WA6027, Perth, Australia;

^6^ National Institute for Data Science in Health and Medicine, Capital Medical University, Beijing, 100069, China.

**Contents of this file**

Text S1 to S3

Figures S1 to S8

Table S1 to Table S3

Text S1. Random forest model building process

Random Forest Regression is a supervised learning algorithm that uses ensemble learning method for regression. Ensemble learning method is a technique that combines predictions from multiple machine learning algorithms to make a more accurate prediction than a single model [1]. We use the randomForest package to fit a random forest model in R to estimate which type of pollutant (gaseous or particulate pollutants) has a greater impact on patients with comorbidities. The basic form of the random forest model is as follows.

$$Outcome \sim Air pollution+Covariates(Meteorology, Time)$$

The $Outcome$ represents the number of daily admissions of patients with combined respiratory and cardiovascular diabetes. $Air pollution$ represents air pollutants of interest including PM_1_, PM_2.5_, PM_10_, SO_2_, NO_2_, CO, O_3_. The $Covariates$ represent the other covariates included in the model, among which the $Meteorology$ include *temperature* and *humidity*, which are included in the model after being transformed into cross-basis matrix using the dlnm package, which controls for lagged effects. $Time$ contains *day of week*, *holiday* and *time trends* to control for time-dependent variables.

The output of the random forest has the following indicators: MSE and Node purity, which represent the size of the impact of the variables on the outcome. Prediction error described as MSE is based on permuting out-of-bag sections of the data per individual tree and predictor, and the errors are then averaged. In the regression context, Node purity is the total decrease in residual sum of squares when splitting on a variable averaged over all trees (i.e. how well a predictor decreases variance). The larger the MSE and Node purity, the greater the impact of the variables on the outcome.

We calculate the mean MSE and Node purity of PM_1_, PM_2.5_, and PM_10_ to represent the effect of particulate pollutants and calculate the average MSE and Node purity of SO_2_, NO_2_, CO, O_3_ representing the effect of gaseous pollutants. We also report Explained variance, the range of values is 0% to 100%. The larger the value means the better the random forest model fits.

There are two important parameters in the random forest model, one is *ntree* and the other is *mtry*. *ntree* refers to the optimal number of decision trees contained in the specified random forest. *mtry* refers to the optimal number of variables used for binomial trees in the specified nodes.

For *ntree*, we first use the default value (*ntree* = 500) for fitting the random forest model, after which we calculate the *ntree* value corresponding to the minimum mean square error (MSE), and if the *ntree* value of the minimum MSE occurs to be less than 500, we select *ntree* = 500 for subsequent analysis. If the *ntree* value of the minimum MSE is equal to 500, the *ntree* value is doubled (*ntree* = 1000) and the model is refitted, and the *ntree* value corresponding to the minimum MSE is calculated, and if the *ntree* value of the minimum MSE is less than 1000, *ntree* = 1000 is chosen for the subsequent analysis. Otherwise, we will repeat the above process until we find the best *ntree* value.

For *mtry*, we also use the default value (*feature*/3) for model fitting, after which we use the *tuneRF()* function in the randomForest package to find the best *mtry* value and refit the model. *tuneRF()* function can continuously change the *mtry* value and calculate the out-of-bag error (OOB Error) estimate. The *mtry* value corresponding to the minimum OOB Error estimate is the best *mtry* value. In order to obtain more accurate results, we set the parameters in the *tuneRF()* function as follows:

*ntreeTry* (number of trees used at the tuning step) is set to 500, *mtryStart* (starting value of mtry) is set to 5, and *stepFactor* (at each iteration, mtry is inflated by this value) is set to 1.5, *improve* (the improvement in OOB error must be by this much for the search to continue) is set to 0.001.

Figure S1 demonstrates the *ntree* selection process. when the outcomes are the daily admissions of patients with CVD-DM and RD-DM, the *ntrees* corresponding to the random forest model are 409 and 498, respectively, neither of which exceeds 500. so we use the model default value of 500 for model fitting.

Figure S2 shows the selection process of *mtry*. the best *mtry* corresponding to the random forest model is 33 when the endpoint is the number of daily admissions for both CVD-DM and RD-DM patients. So we use the best *mtry* value (*mtry = 33*) for fitting the random forest model in addition to the default *mtry* value (*mtry = 16*) of the model.

Text S2. Causal association modeling process

Due to the limitations of quasi-experimental methods such as instrumental variables, double difference, and breakpoint regression in the application of observational studies [2, 3]. In recent years, an increasing number of studies have resorted to negative control (NC) methods to detect or control for unobserved confounders [4, 5].

The Negative-Control Exposure in Time-Series Study (NCETS) method is an improved causal inference method proposed by Xue et al. in 2020 [6]. The method addresses the widespread data incompleteness in big health data and creates an original method to eliminate all confounding factors and thus accurately infer the causal effect size of exposure on outcome when only exposure and outcome are available, which has important evidence-based medical implications for the objective and accurate evaluation of the effect of environmental exposure on health/disease.

Causal diagram in Figure S3 were used to illustrate the NCETS model building process [6]. *X_t-h_* denotes the observed value of exposure *X* at time *t-h* (e.g., PM_2.5_ at time *t-h*); *Y_t_* indicates the observed value of outcome *Y* occurring at time *t* (e.g., admissions of CVD-DM patients at time *t*); *X_t+h_* at time *t+h* can be observed as a negative control exposure variable; *U_t-h_*, *U_t_*, *U_t+h_* are confounding for *t-h*, *t*, and *t+h*, respectively (containing known and unknown confounding). From Figure 3, future exposure *X_t+h_* does not affect past disease outcome *Y_t_*, and *Y_t_* does not affect *X_t+h_*, which means there is no bidirectional causality. In environmental epidemiological studies, it is easy to determine that bidirectional causality (*Y_t_* → *X_t+h_*) does not exist (e.g., PM_2.5_ is the cause of hospital admissions, and hospital admissions are never the cause of PM_2.5_). Therefore, the above modeling assumption of "no reverse causality" is reasonable in most cases.

In our study, *X_t-h_* is the concentration of air pollutants (including PM_1_, PM_2.5_, PM_10_, SO_2_, NO_2_, CO, O_3_) at time *t-h*. *Y_t_* is the number of comorbid admissions (including total respiratory, cardiovascular disease and subtypes) at time *t*. *U* is other important confounding variables (including temperature, humidity and time trends). At this point, air pollutants (*X_t+h_*) can be reintroduced at *t+h* after the onset of the *Y_t_* outcome, and the NCETS model can be constructed to identify and estimate the causal effect of air pollutant concentrations on the risk of hospital admissions for comorbid patients.

We used the NCETS package, freely available on GitHub, developed by Xue et al. to estimate the size of the causal effect of air pollution on patients with comorbidities. Considering that our outcomes are continuous variables, we fit the causal effects model using the *ncets_con()* function from the NCETS package. Two methods of estimating confidence intervals are provided in the *ncets_con()* function. "normal" means that the confidence interval is obtained by calculating the covariance matrix; "bootstrap" means that the confidence interval is calculated by the resampling method. To verify the stability of our results, we use both methods to calculate confidence intervals for the causal effects. The output from *ncets_con()* function is called *causal effect estimator,* the higher the value means the stronger the causal effect.

Text S3. Data download steps

Air pollutant data of NO_2_, SO_2_ and CO data are available at https://quotsoft.net/air/, which operation steps include 3 steps.

First, we should enter the URL in the browser with the link https://quotsoft.net/air/.


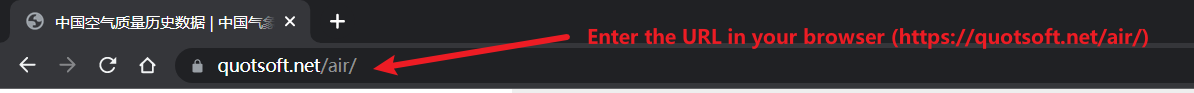


Second, click on the Beijing air quality data download link, which has been marked with a box in the chart below.


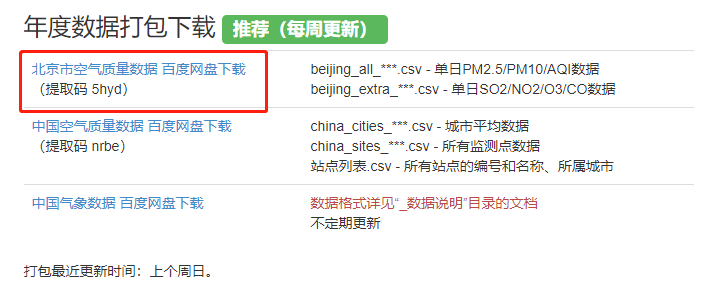


Third, due to the data downloaded in the previous step is a compressed package, then unzip it and select the data needed to analyze.


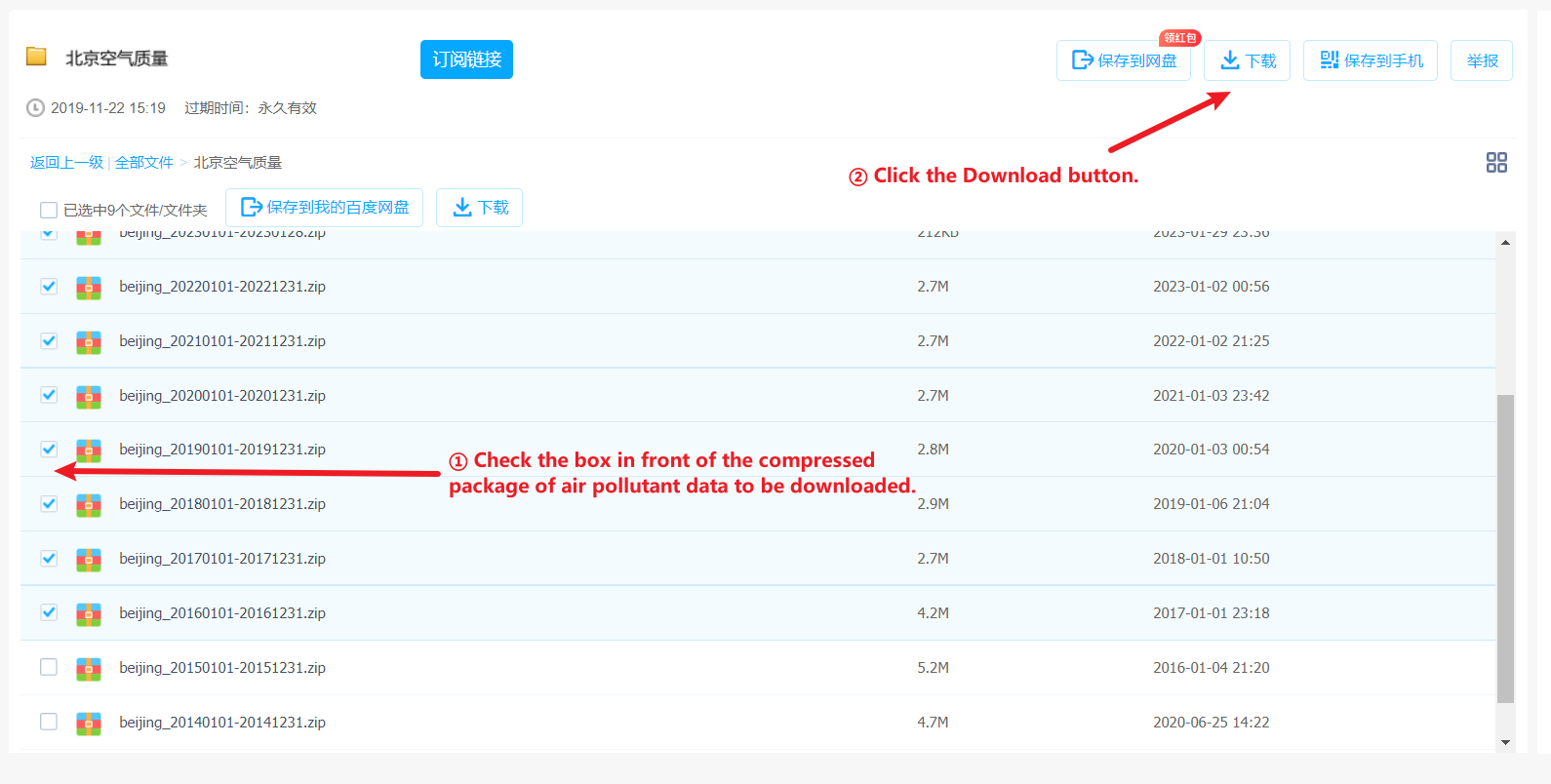


Meteorological data are available at http://data.cma.cn, which operation steps include 6 steps.

First, we should enter the URL in the browser with the link http://data.cma.cn.


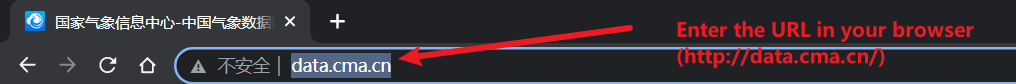


Second, register your account by email and log in.


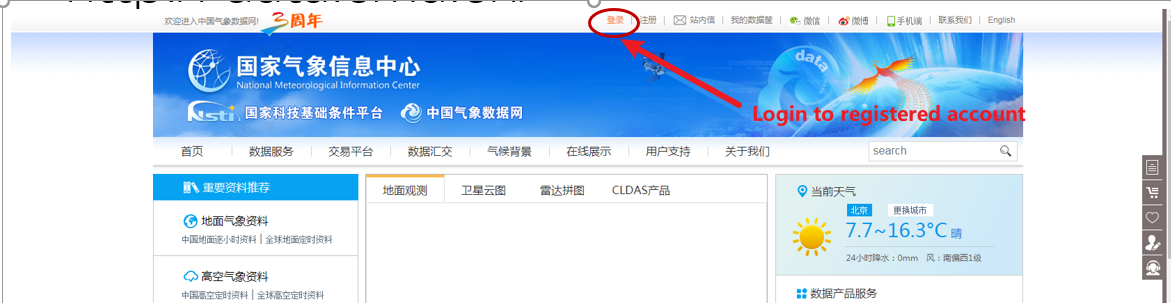


Third, click the Shared Directory option under Data Services.


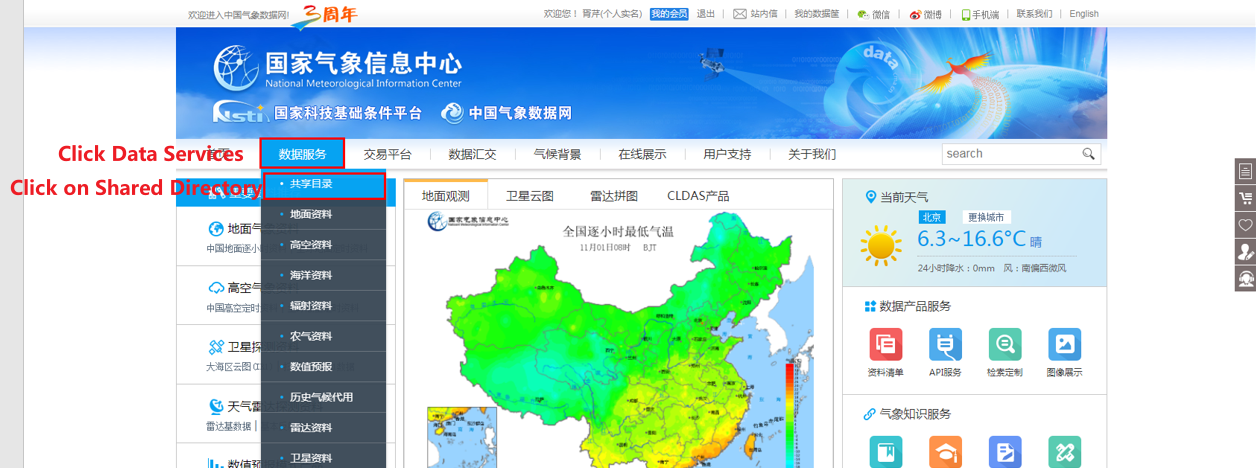


Fourth, click on the option China Ground Weather Station Hour-by-Hour Observations.


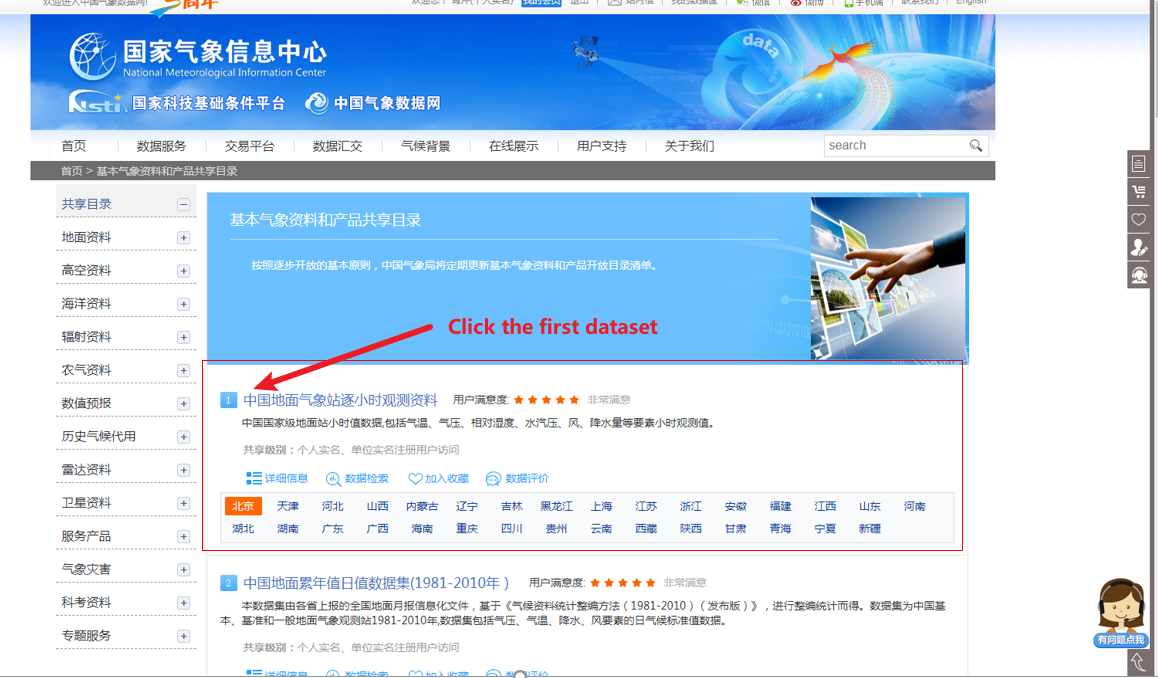


Fifth, click on the Data Retrieval option.


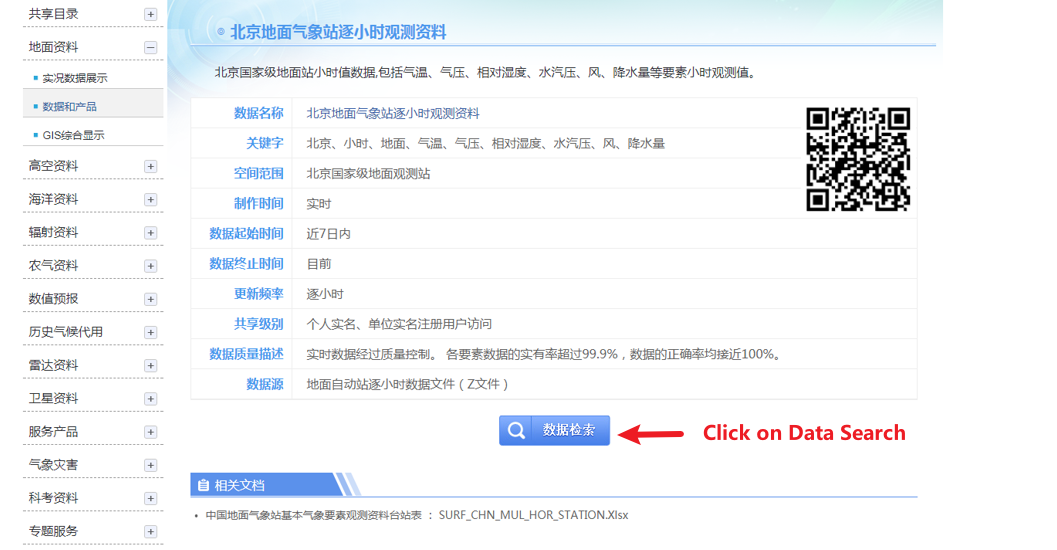


Sixth, select the date, area and meteorological variables, and then download the data.


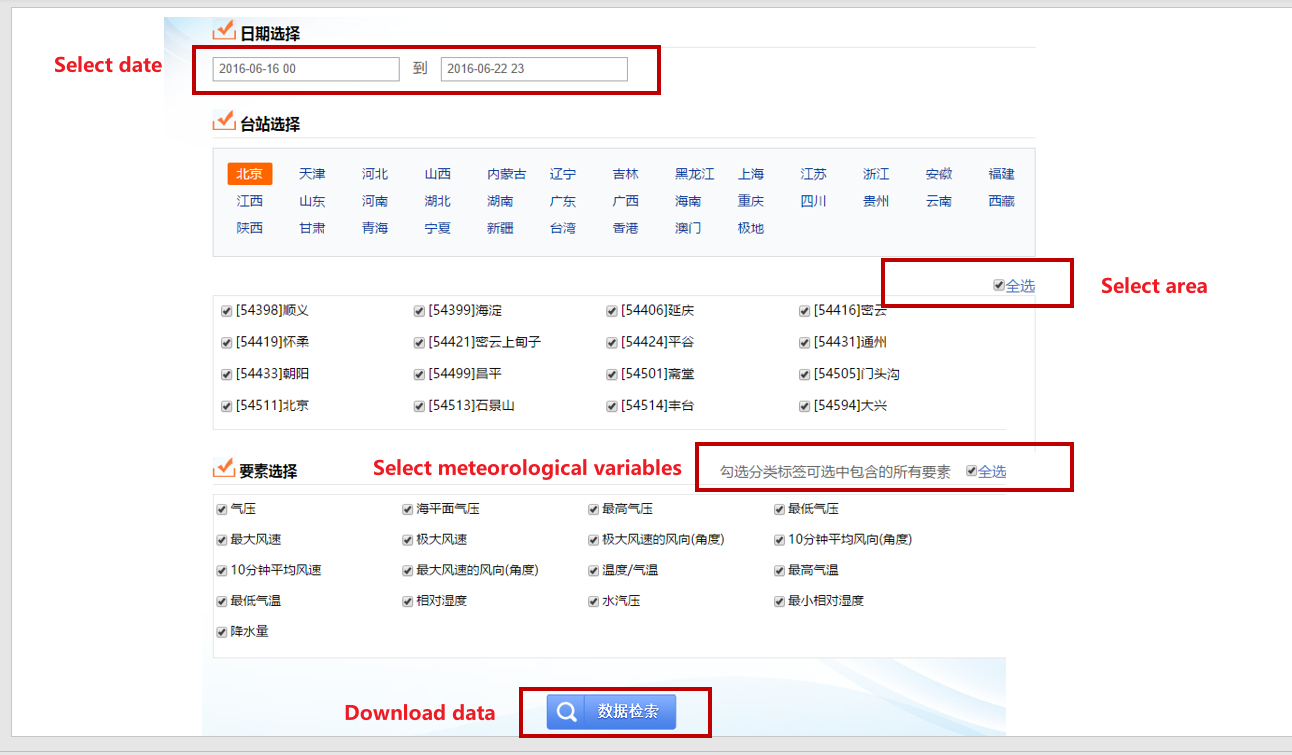


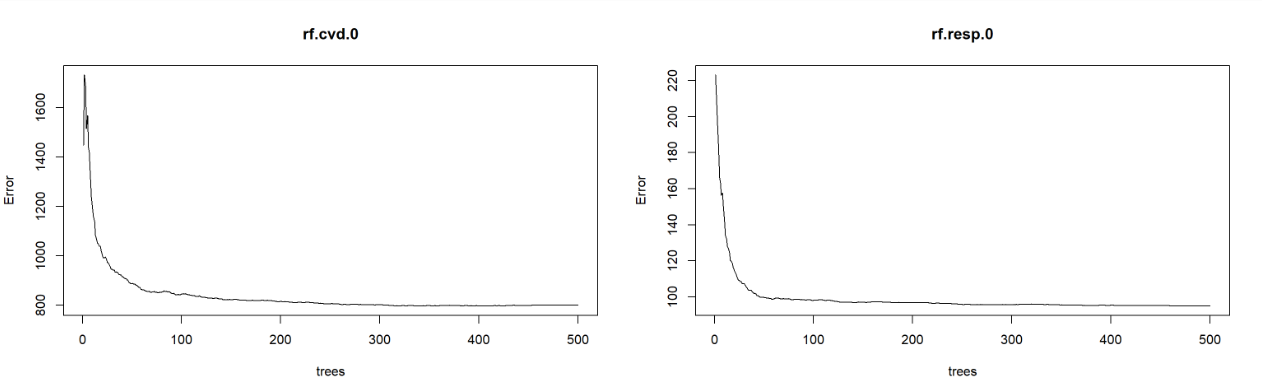


**Figure S1 The minimum mean square error corresponding to different ntree.**

Note: The outcome of the left panel is CVD-DM, and when the MSE is minimal the corresponding ntree is 409. The outcome of the right panel is RD-DM, and when the MSE is minimal the corresponding ntree is 498.


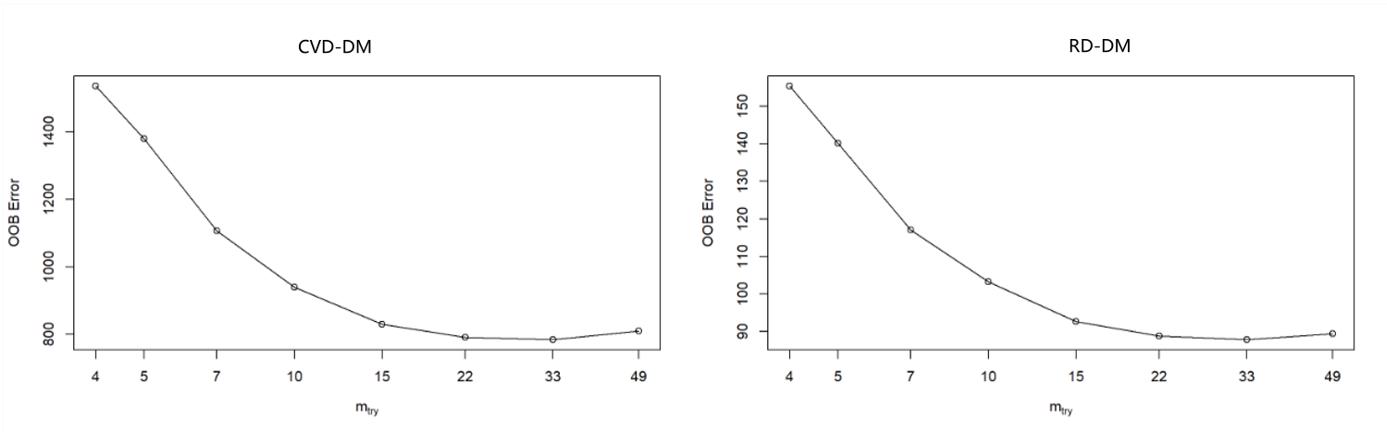


**Figure S2 The minimum OOB Error corresponding to different mtry.**

Note: The outcome of the left panel is CVD-DM, and when the OOB Error is minimal the corresponding mtry is 33. The outcome of the right panel is RD-DM, and when the OOB Error is minimal the corresponding mtry is 33.


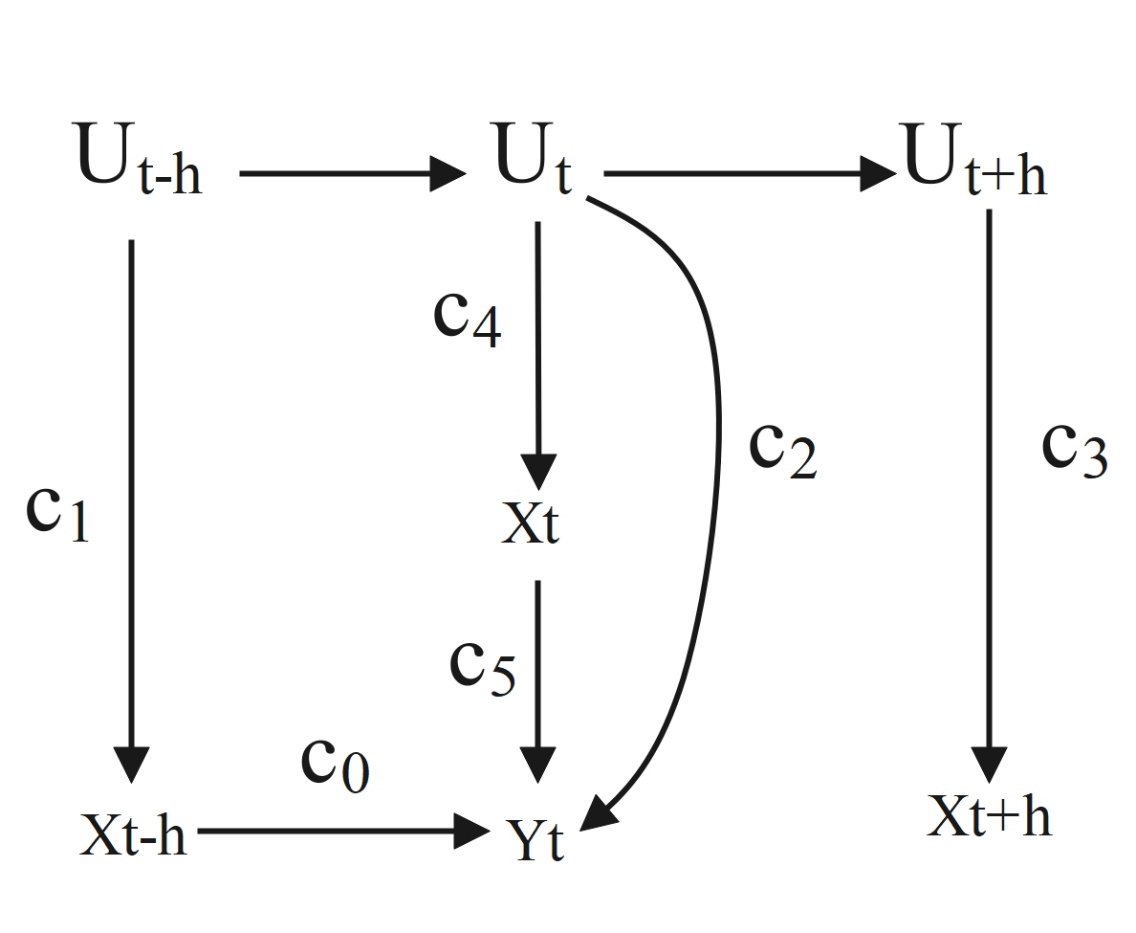


**Figure S3 Causal schematic based on NCETS model.**

Note: c_0_, c_1_, c_2_, c_3_, c_4_, c_5_ represent the corresponding effect magnitude.


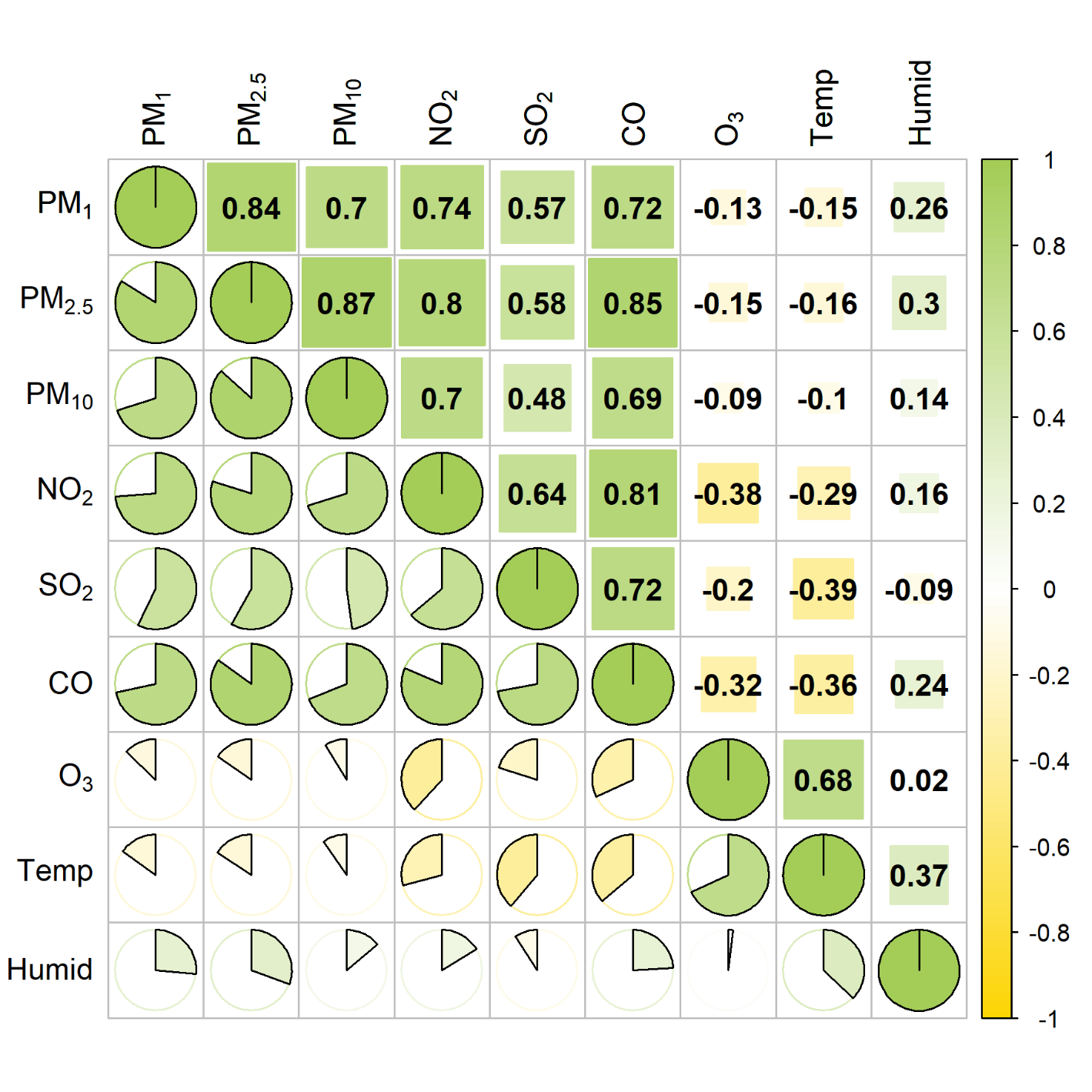


**Figure S4 Spearman correlation matrix of air pollution and meteorological variables.**


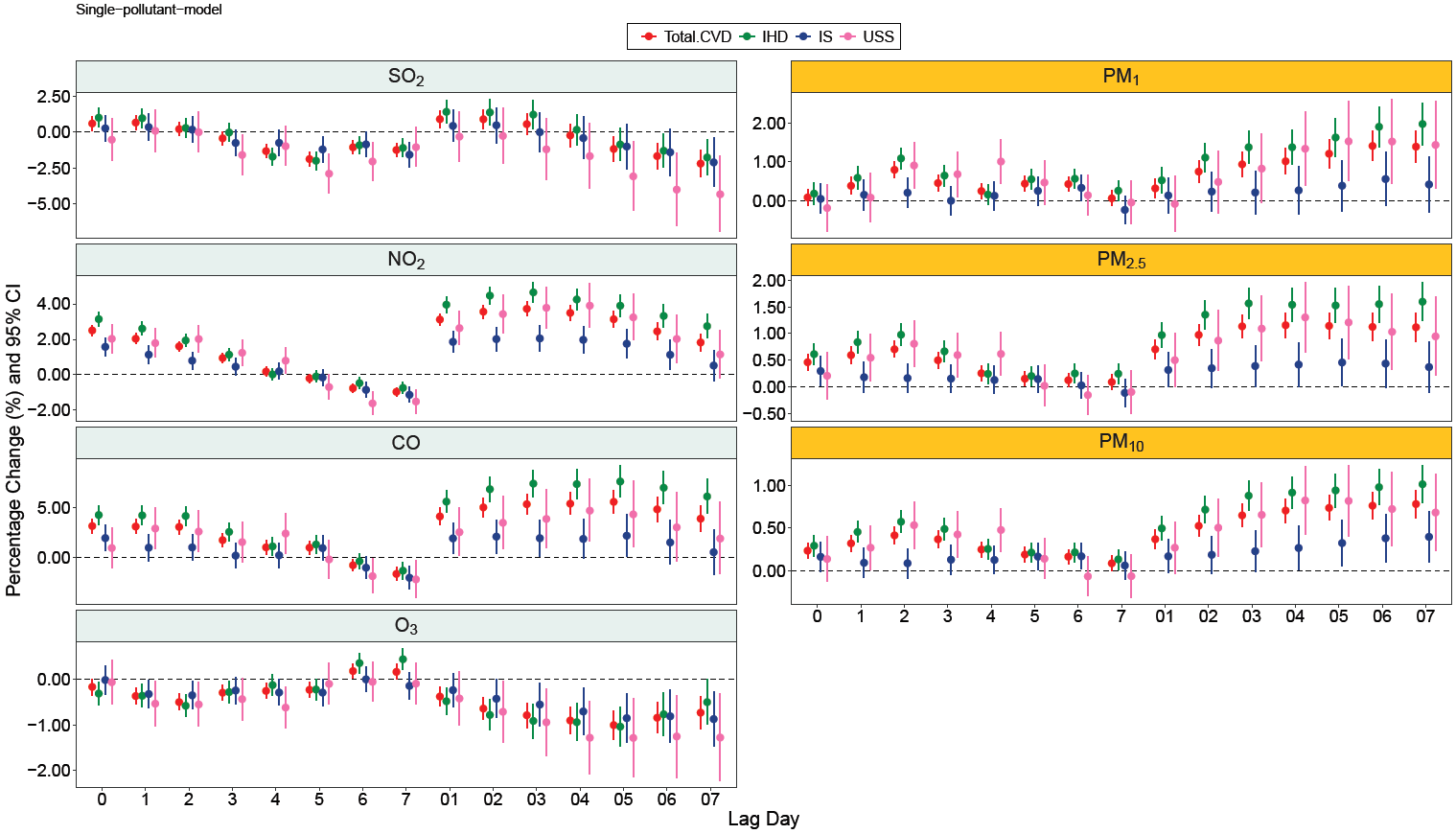


**Figure S5 The percentage change for CVD-DM patients in 7 air pollutants with different lag structure in single model (only adjusted temperature and relative humidity).** Note: Percentage Change for PM_2.5_, PM_10_, SO_2_, NO_2_ and O_3_ were per 10 ug/m^3^ increase and 1 mg/m^3^ for CO.


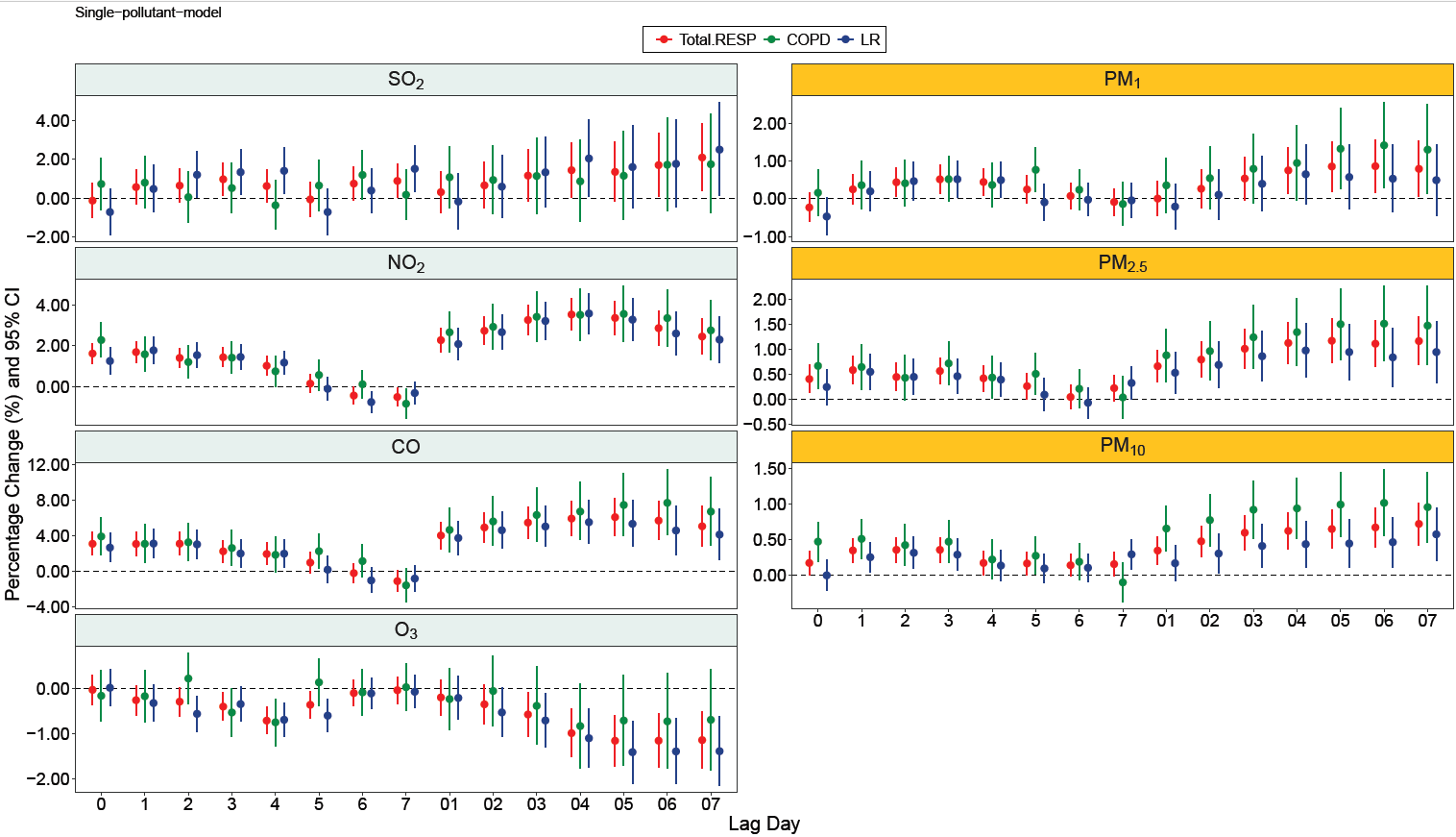


**Figure S6 The percentage change for RD-DM patients in 7 air pollutants with different lag structure in single model (only adjusted temperature and relative humidity).** Note: Percentage Change for PM_2.5_, PM_10_, SO_2_, NO_2_ and O_3_ were per 10 ug/m^3^ increase and 1 mg/m^3^ for CO..


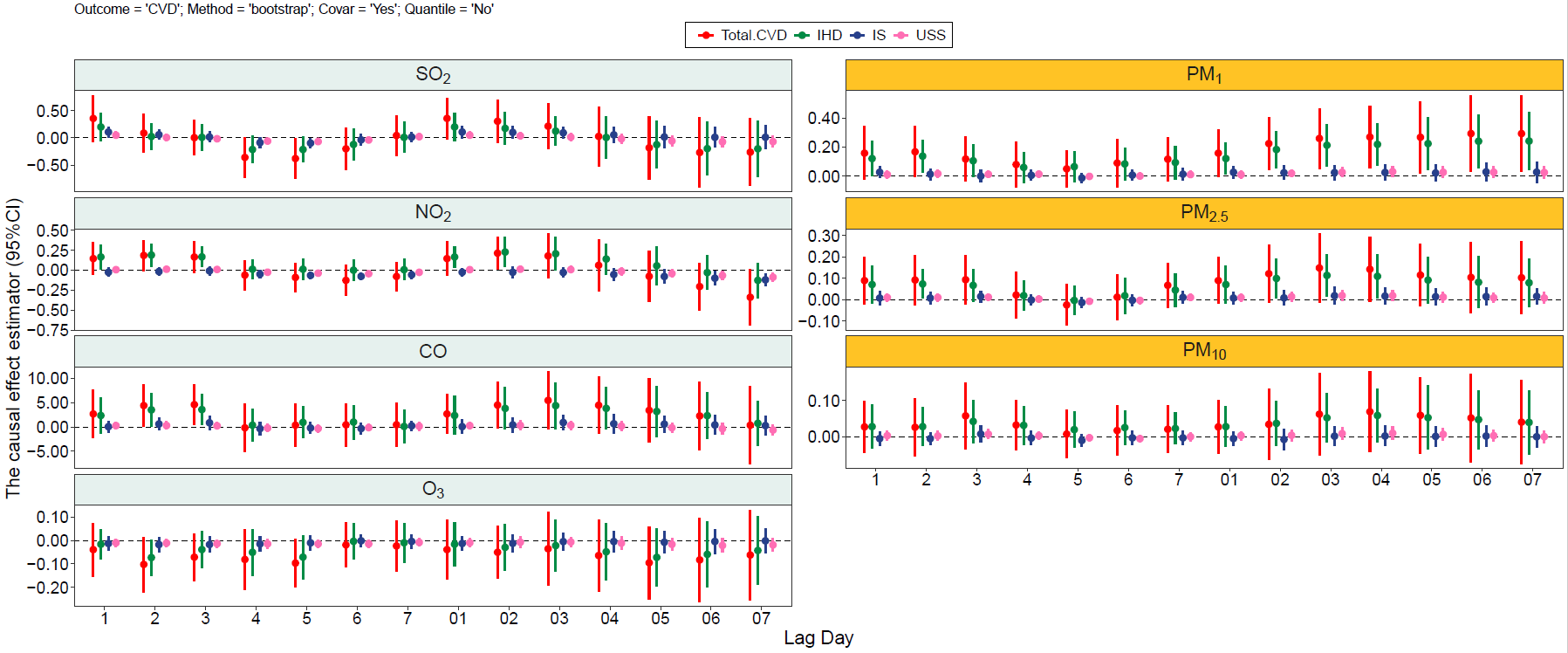


**Figure S7 Causal effects of different air pollutants for total and specific CVD-DM patients using “bootstrap” method.**


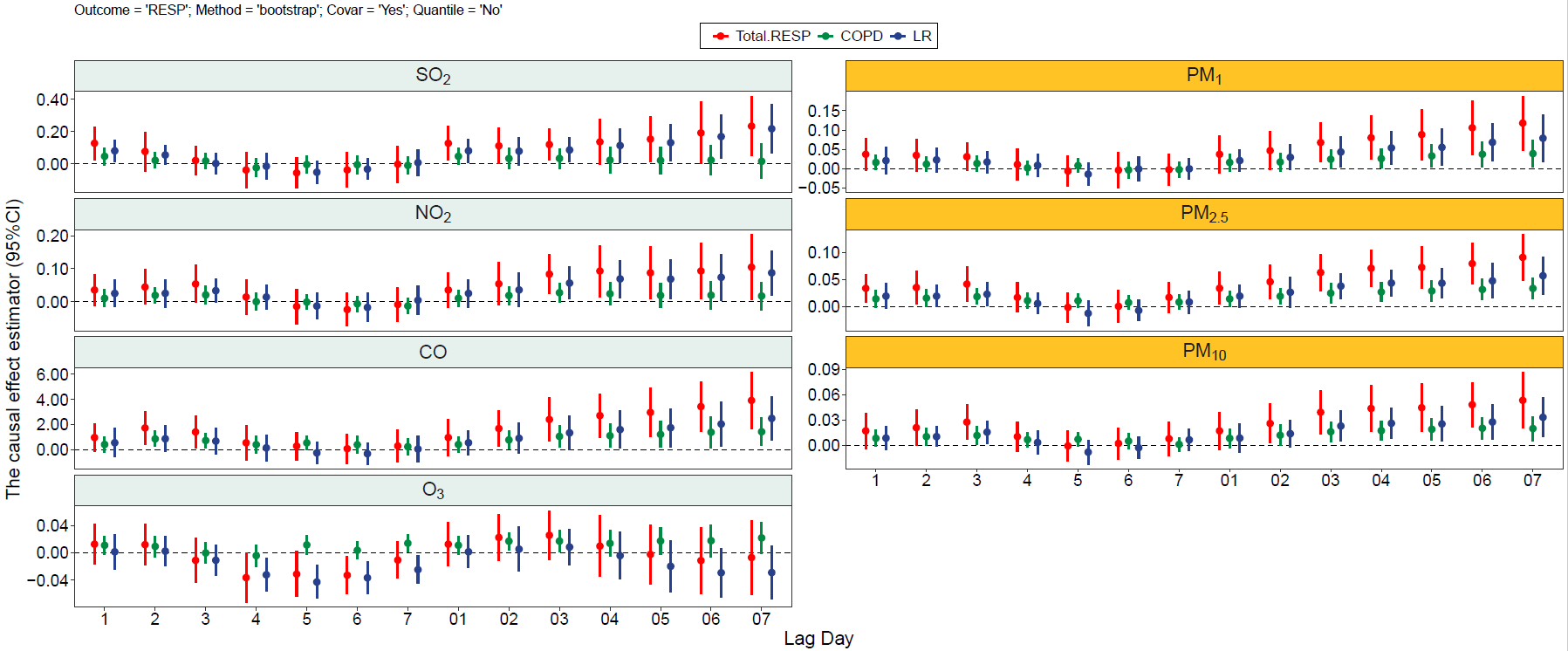


**Figure S8 Causal effects of different air pollutants for total and specific RD-DM patients using “bootstrap” method.**

**Table S1 E value result for particulate matter air pollution in different CVD and RD patients.**

|  |  | RR* | E value | Stable |
| --- | --- | --- | --- | --- |
| PM_1_ | Total CVD | 1.01 | 1.12 | Yes |
|  | IHD | 1.02 | 1.14 | Yes |
|  | IS | 1.00 | 1.06 | Yes |
|  | USS | 1.01 | 1.14 | Yes |
|  | Total RD | 1.01 | 1.08 | Yes |
|  | COPD | 1.01 | 1.10 | Yes |
|  | LR | 1.01 | 1.09 | Yes |
| PM_2.5_ | Total CVD | 1.01 | 1.12 | Yes |
|  | IHD | 1.02 | 1.14 | Yes |
|  | IS | 1.00 | 1.06 | Yes |
|  | USS | 1.01 | 1.14 | Yes |
|  | Total RD | 1.01 | 1.12 | Yes |
|  | COPD | 1.01 | 1.13 | Yes |
|  | LR | 1.01 | 1.12 | Yes |
| PM_10_ | Total CVD | 1.01 | 1.09 | Yes |
|  | IHD | 1.01 | 1.10 | Yes |
|  | IS | 1.00 | 1.06 | Yes |
|  | USS | 1.01 | 1.10 | Yes |
|  | Total RD | 1.01 | 1.11 | Yes |
|  | COPD | 1.01 | 1.08 | Yes |
|  | LR | 1.01 | 1.09 | Yes |

*: All RR are greater than 1, because we calculate E value by taking the inverse of RR less than 1.

**Table S2 E value result for gaseous air pollution in different CVD and RD patients.**

|  |  | RR* | E value | Stable |
| --- | --- | --- | --- | --- |
| SO_2_ | Total CVD | 1.00 | 1.07 | Yes |
|  | IHD | 1.01 | 1.09 | Yes |
|  | IS | 1.00 | 1.04 | Yes |
|  | USS | 1.01 | 1.12 | Yes |
|  | Total RD | 1.02 | 1.18 | Yes |
|  | COPD | 1.02 | 1.15 | Yes |
|  | LR | 1.00 | 1.07 | Yes |
| NO_2_ | Total CVD | 1.04 | 1.25 | Yes |
|  | IHD | 1.05 | 1.28 | Yes |
|  | IS | 1.02 | 1.18 | Yes |
|  | USS | 1.04 | 1.26 | Yes |
|  | Total RD | 1.03 | 1.22 | Yes |
|  | COPD | 1.04 | 1.25 | Yes |
|  | LR | 1.04 | 1.24 | Yes |
| CO | Total CVD | 1.05 | 1.27 | Yes |
|  | IHD | 1.07 | 1.34 | Yes |
|  | IS | 1.02 | 1.15 | Yes |
|  | USS | 1.05 | 1.27 | Yes |
|  | Total RD | 1.06 | 1.33 | Yes |
|  | COPD | 1.06 | 1.32 | Yes |
|  | LR | 1.06 | 1.31 | Yes |
| O_3_ | Total CVD | 1.00 | 1.05 | Yes |
|  | IHD | 1.00 | 1.07 | Yes |
|  | IS | 1.00 | 1.01 | Yes |
|  | USS | 1.00 | 1.05 | Yes |
|  | Total RD | 1.00 | 1.02 | Yes |
|  | COPD | 1.00 | 1.00 | Yes |
|  | LR | 1.00 | 1.05 | Yes |

*: All RR are greater than 1, because we calculate E value by taking the inverse of RR less than 1.

**Table S3 The contribution of gaseous and particulate pollutants in random forest model for cause specific CVD and RD patients with DM with different mtry value.**

| mtry* | Outcome | Pollution type | MSE^c^ | Node Purity | Explained variance |
| --- | --- | --- | --- | --- | --- |
| 33 | IHD-DM | Gaseous^a^ | 10.44 | 27130.06 | 85% |
|  |  | Particulate^b^ | 5.39 | 17629 |  |
|  | IS-DM | Gaseous | 10.11 | 8289.57 | 80% |
|  |  | Particulate | 7.72 | 4528.2 |  |
|  | USS-DM | Gaseous | 10.74 | 2583.79 | 69% |
|  |  | Particulate | 9.38 | 2117.72 |  |
|  | COPD-DM | Gaseous | 12.42 | 1763.3 | 66% |
|  |  | Particulate | 10.65 | 1274.9 |  |
|  | LR-DM | Gaseous | 9.69 | 5711.33 | 85% |
|  |  | Particulate | 6.36 | 2958.45 |  |
| 16 | IHD-DM | Gaseous | 12.13 | 53002.56 | 85% |
|  |  | Particulate | 7.67 | 28252.7 |  |
|  | IS-DM | Gaseous | 13.16 | 11362.95 | 79% |
|  |  | Particulate | 8.62 | 7248.67 |  |
|  | USS-DM | Gaseous | 11.78 | 3008.96 | 70% |
|  |  | Particulate | 8.96 | 2470.77 |  |
|  | COPD-DM | Gaseous | 11.14 | 1922.52 | 65% |
|  |  | Particulate | 10.1 | 1471.57 |  |
|  | LR-DM | Gaseous | 12.24 | 10763.91 | 84% |
|  |  | Particulate | 9.17 | 6025.25 |  |

a: SO_2_, NO_2_, CO, O_3_ are included in gaseous pollution; b: PM_1_, PM_2.5_, PM_10_ are included in particulate pollution; c: Mean Square Error.

*: The optimal mtry is the mtry corresponding to the minimal Out Of Bag Error in model (mtry = 33), and the default mtry is the number of variables divided by 3 (mtry = 16)
